# Supplementary material for: Frequency and Predictors of Adolescent Worry for School Gun Violence In the United States: Findings from a Nationally Representative Study
Source: medRxiv. 2025 Feb 20:2025.02.18.25322472. Preprint. [Version 1] doi: 10.1101/2025.02.18.25322472 (PMC11875268; doi:10.1101/2025.02.18.25322472)
Supplement: 1 [file NIHPP2025.02.18.25322472V1-supplement-1.pdf]

medRxiv preprint doi: https://doi.org/10.1101/2025.02.18.25322472; this version posted February 20, 2025. The copyright holder for this preprint (which was not certified by peer review) is the author/funder, who has granted medRxiv a license to display the preprint in perpetuity.
It is made available under a CC-BY 4.0 International license .

Supplemental References

1. Chen TJ, Whitfield GP, Watson KB, et al. Awareness and Knowledge of the Physical Activity Guidelines for Americans, 2nd Edition. *J Phys Act Health*. Aug 1 2023;20(8):742-751. doi:10.1123/jpah.2022-0478
2. Cox CG, Davis M, Grill J, Roberts J. US adults' likelihood to participate in dementia prevention drug trials: Results from the national poll on healthy aging. *The journal of prevention of Alzheimer's disease*. 2023;10(1):34-40.
3. Kiviniemi MT, Orom H, Hay JL, Waters EA. Prevention is political: political party affiliation predicts perceived risk and prevention behaviors for COVID-19. *BMC public health*. 2022;22(1):298.
4. Scherer LD, Lewis CL, McCaffery K, et al. Mammography screening preferences among screening-eligible women in their 40s: A National US survey. *Annals of Internal Medicine*. 2024;177(8):1069-1077.
5. Semenza DC, Ziminski D, Anestis MD. Physical intimate partner violence and emotional harm in five US states. *Journal of interpersonal violence*. 2024;39(9-10):2344-2368.
